# An unusual cause of a haemothorax following pacemaker implantation: A case report

**DOI:** 10.1093/ehjcr/ytac185

**Published:** 2022-05-01

**Authors:** Christopher E. D. Saunderson, Andrew J. Hogarth, Sotiris Papaspyros, Costa Tingerides, Muzahir H. Tayebjee

**Affiliations:** 1 Department of Cardiology, Yorkshire Heart Centre, Leeds General Infirmary, Leeds Teaching Hospitals NHS Trust, Leeds, UK; 2 Department of Diagnostic and Interventional Radiology, Leeds General Infirmary, Leeds Teaching Hospitals NHS Trust, Leeds, UK; 3 Department of Cardiology, Leeds Institute of Cardiovascular and Metabolic Medicine, University of Leeds, Leeds, UK

**Keywords:** Pacemaker, haemothorax, lead perforation, complication, intercostal artery, case report

## Abstract

**Background:**

Haemothoraces are a reported but extremely rare complication of pacemaker implantation. Haemothoraces can be a consequence of lead perforation through the right ventricle (RV) and pericardium into the pleural space, direct lung or vascular injury during access.

**Case summary:**

A 72-year-old woman presented 24 h after a pacemaker implantation with chest pain and shortness of breath. Computed tomography of the chest confirmed perforation of the RV lead into the left pleural cavity with a large left sided haemothorax. Following percutaneous drainage of the left sided haemothorax, the patient became haemodynamically unstable necessitating emergent sternotomy. During surgery, the extra-cardiac portion of the pacing lead was cut, the RV repaired and a large haematoma evacuated from the left pleural space. Despite this, the patient remained hypotensive, and further exploration showed a bleeding intercostal artery that had been lacerated by the pacing lead. This was treated by electrocautery, and the patient’s haemodynamic status improved. The RV lead remnant was removed transvenously via the subclavian vein, and the patient was left with a single chamber atrial pacemaker.

**Discussion:**

Prompt recognition of RV lead perforation and its associated sequalae, often utilising multi-modality imaging, is vital to enable transfer to a centre with cardiac surgical expertise. In this case, the perforating RV lead lacerated an intercostal artery, and this was only identified at the time of surgery. In order to minimize the risk of perforation, multiple fluoroscopic views should be used, and care should be taken during helix deployment.

**Learning points**

- Haemothorax without pneumothorax should alert the clinician to cardiac perforation as opposed to a haemopneumothorax which is generally due to inadvertent vascular injury during access.
- Multi-modality imaging can help confirm the presence of a haemothorax, identify perforation or vascular injury to aid decision-making on subsequent management.
- Management of lead perforation should be undertaken in centres with onsite cardiac surgical expertise.

## Introduction

A haemothorax is an extremely rare complication of pacemaker implantation. Haemothoraces can be secondary to lead perforation through the right ventricle (RV) and pericardium into the pleural space, direct lung or vascular injury during access.^1–3^ Cardiac perforation as result of pacing leads is uncommon but can be life-threatening.^4^ Perforations most commonly occur at the time of the procedure or within the 24 h after implantation, but delayed perforations have also been recognized. Here, we describe a case of a large haemothorax from RV perforation and laceration of an intercostal artery.

## Timeline

**Table:** 

Initial presentation (Day 0)	A 72-year-old female undergoes an uneventful pacemaker implant for paroxysmal atrial fibrillation with post termination pauses.
Day 1	Patient develops chest pain and shortness of breath.
Day 2	Device interrogation and CT chest confirm RV lead perforation with large volume haemothorax.
	Patient develops haemorrhagic shock necessitating emergency sternotomy to remove the RV lead and repair a severed intercostal artery.
Day 23	Admission is complicated by bilateral pulmonary emboli.
Day 34	Patient discharged.
Day 44	Patient recovering and pacemaker functioning appropriately on clinic review.

## Case presentation

**Figure 1:**
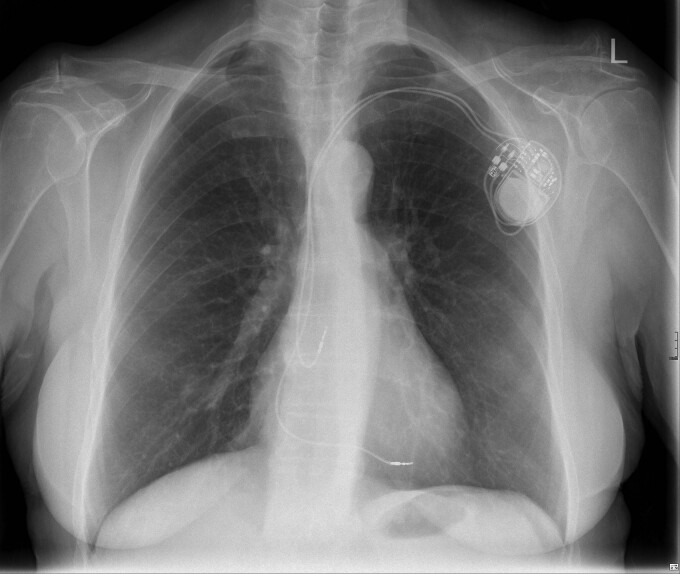
Chest radiograph performed 2 h post procedure.

A 72-year-old woman with paroxysmal atrial fibrillation and symptomatic 6 s post termination pauses was scheduled for an outpatient pacemaker. She had previously undergone catheter ablation for Wolff-Parkinson-White syndrome and atrial flutter and was known to have primary biliary cirrhosis. The patient was taking bisoprolol 1.25 mg and ursodeoxycholic acid. She was not on anti-coagulation due to a remote variceal bleed although recent endoscopy did not show any evidence of varices. The patients CHADVASC score was 2, so she had been referred to the haematology clinic for consideration of anti-coagulation. A dual chamber pacemaker was inserted via the extra-thoracic subclavian vein without immediate complication. Active fixation pacemaker leads (Medtronic CapSureFix Novus #5076: 52 cm and 45 cm) were placed in the RV apex and right atrial appendage, respectively, using fluoroscopic screening in the posterior-anterior projection. The patient was in atrial fibrillation with ventricular rates up to 150 bpm during the procedure and spontaneously converted to sinus rhythm prior to leaving the catheter laboratory. Ventricular lead pacing parameters at implant and at a second check prior to leaving the catheter laboratory were as follows: sensing 9 mV, lead impedance 1200Ω and pacing threshold 0.5 V at 0.4 ms. On return to the ward the patients’ observations were as follows: blood pressure (BP) 152/89 mmHg, heart rate 67 bpm, respiratory rate 19 breaths/minute, and oxygen saturations 96% on room air. Post-procedure pacing checks and chest radiograph (*Figure 1*) were satisfactory, and the patient was discharged home the same day.

**Figure 2:**
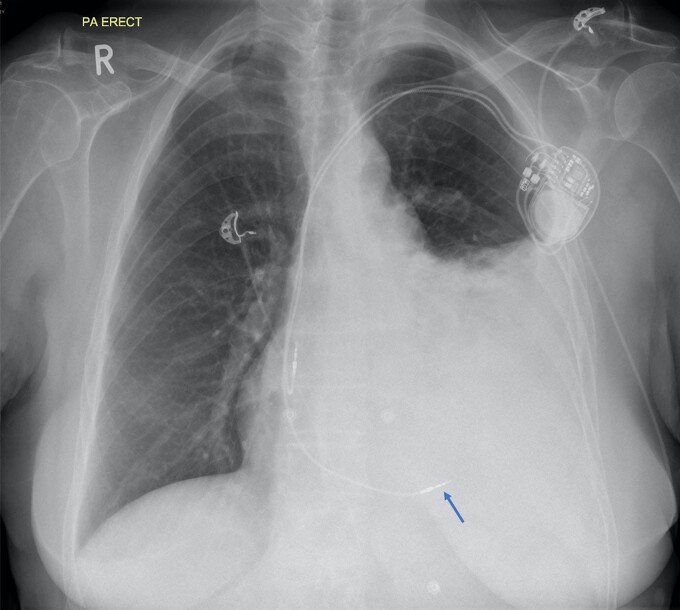
Chest radiograph performed 24 h post procedure demonstrating a new large left pleural effusion and atypical position of right ventricular lead (arrow).

She presented 24 h later to her local accident and emergency department with a 12-hour history of sharp left-sided chest pain and shortness of breath. A chest radiograph performed at the local hospital demonstrated a new large volume left-sided pleural effusion with RV lead displacement (*Figure 2*).

**Figure 3:**
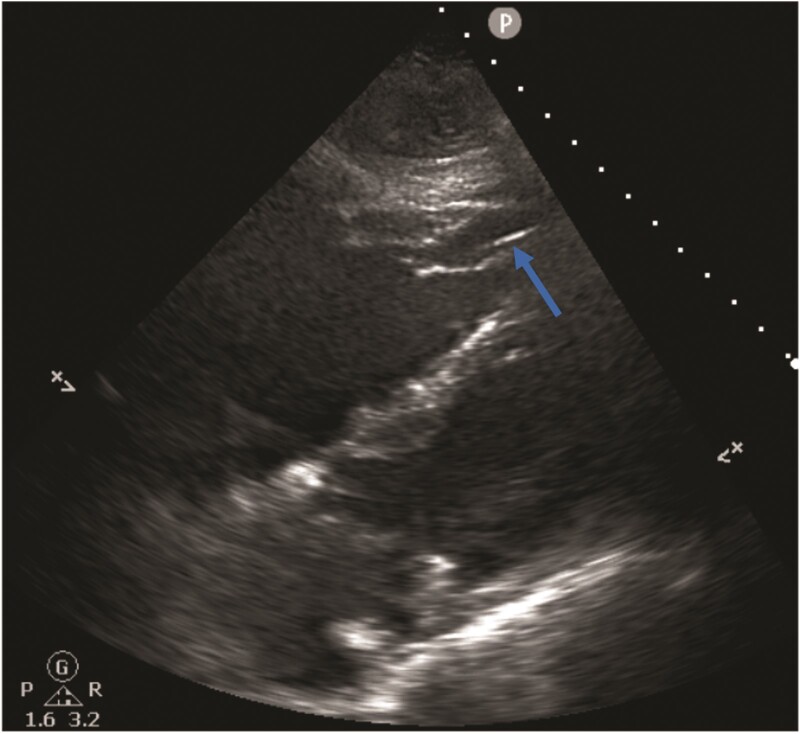
Subcostal view on echocardiogram demonstrating part of the pacing lead in the right ventricular apex (arrow). No pericardial effusion is seen.

RV lead perforation was suspected, and pacemaker interrogation revealed no sensing or capture at high output (5 volts at 1.0 milliseconds). The patient was subsequently transferred to the implanting centre for ongoing management. On examination, the patient was comfortable at rest. She had a body mass index of 22.6 kg/m^2^. Blodd pressure was 109/67 mmHg with a heart rate of 75 bpm in sinus rhythm. Respiratory rate was 17 breaths/minute with oxygen saturations of 97% on room air. Chest examination revealed no murmur or rub, but there were diminished breath sounds at the left base. Her haemoglobin fell from 138 g/L (normal 115–160 g/L) prior to device implantation to 109 g/L. Renal function was normal (Creatinine 58 umol/L; normal 49–90 umol/L). There was no pericardial effusion on echocardiography (*Figure 3*).

**Figure 4:**
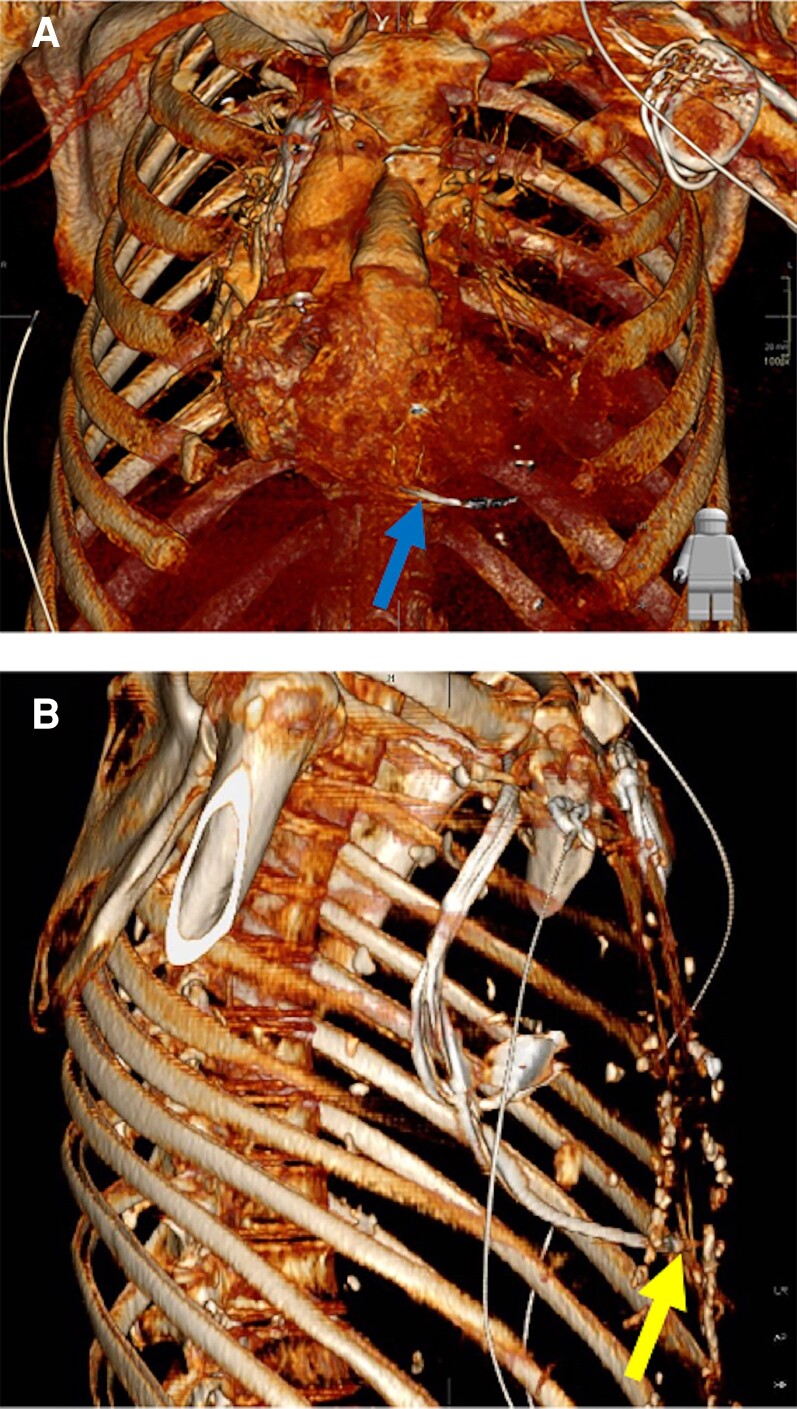
Three-dimensional computed tomography reconstruction (volume rendering) demonstrating the pacing lead perforating through the right ventricle (panel A arrow) and lying in close proximity to anterior intercostal vessels (panel B arrow)*. *Image produced using IMPAX volume viewing 4.0; Clinapps 7.0.282.0 (Agfa healthcare)

Computed tomography (CT) pulmonary angiography of the chest was performed which showed a large left-sided haemothorax causing mediastinal shift and a tiny pericardial effusion. The RV pacing lead was seen to perforate through the cardiac apex and across the left pleural space (*Figure 4*). No active site of bleeding was identified on the CT angiography.

**Figure 5:**
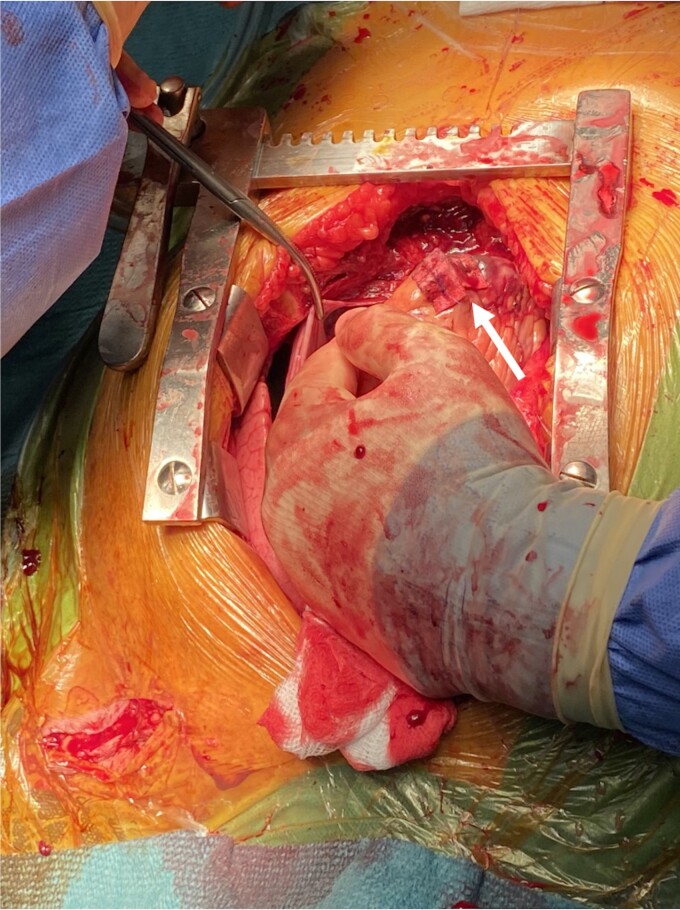
Patch repair of right ventricular apex after removal of pacing lead (arrow). The surgeon was manually compressing the intercostal artery.

The case was discussed in a multi-disciplinary setting with the cardiac surgical team. Given the patients age, co-morbidities, and haemodynamic stability, a decision was made to first insert a chest drain and continue to monitor the patient. If the patient remained stable following aspiration of the haemothorax, it was decided to attempt transvenous lead re-position in the cardiac catheter laboratory with surgical back-up. After 2 h, 1500 mL of frank blood had been drained, and the patient now was pale, tachycardic (140 bpm in atrial fibrillation), and hypotensive (BP 70/40 mmHg). Despite fluid resuscitation, the patient’s clinical condition continued to deteriorate and she was taken for emergency surgery. A median sternotomy was performed, and the pacing lead was found to have perforated the RV apex into the left pleural cavity without causing pericardial bleeding. The external portion of the RV lead was cut, and the intra-cardiac portion was removed transvenously. The RV wall was repaired and haemothorax evacuated (*Figure 5*). Despite this, the patient remained hypotensive, and further exploration identified ongoing bleeding into the pleural cavity from a lacerated intercostal artery. Electrocautery was applied to the bleeding vessel. The atrial lead was left in situ, and the patient was left with a single chamber pacemaker.

Following surgery, the patient remained in atrial fibrillation but made steady progress although her admission was complicated by the development of bilateral pulmonary emboli on Day 23. She was commenced on warfarin and was discharged 2 weeks later. Given the patient remained in rate controlled atrial fibrillation after protracted admission and had intact atrio-ventricular nodal function, no further attempts were made to position a ventricular lead. At follow-up in the device clinic, she remains in atrial fibrillation but is recovering well with normal device function.

## Discussion

Cardiac perforation following pacemaker implantation, although rare, is potentially life-threatening. The incidence of RV perforation following pacemaker implantation is reported to be 0.1–0.8% with the majority presenting at the time of implant or within the first 24 h.^4^ Presentation may depend on the location of the displaced lead with tamponade, pacing malfunction (such as loss of capture), and diaphragmatic or chest muscle stimulation all having been reported.^5^ Haemothoraces are an extremely rare complication of pacemaker implantation. Patients may present with chest pain, dyspnoea, tachycardia, and even hypotension if there is associated haemorrhagic shock.^2^ Ipsilateral haemothorax can be a consequence of ventricular perforation, inadvertent vascular injury whilst obtaining access or direct lung injury.^1,3^ The presence of haemothorax without pneumothorax should alert the clinician to potential cardiac perforation as a cause rather than vascular injury sustained whilst attempting venous access.

This case highlights the need for multi-disciplinary management in certain pacing complications. Early recognition of the haemothorax and potential lead perforation was critical in this case as the patient could be immediately transferred to the implanting centre with on-site cardiac surgical expertise. Furthermore, the availability of immediate multi-modality imaging meant that the presence of a large haemothorax with RV lead perforation could be confirmed with exclusion of co-existent pericardial effusion. Computed tomography angiography should be considered in these cases. An arterial and delayed phase should be performed as a minimum to accurately identify active bleeding and determine the source. Management of lead perforation depends on the patient’s symptoms, haemodynamic status, and presence of any significant pericardial or pleural effusion although lead extraction is usually mandated.^6,7^ Current Heart Rhythm Society guidelines recommend that extraction should be performed by a collaborative lead extraction team, with the need for an on-site cardiac surgical team.^7^ The importance of this is clearly highlighted in this case as the patient rapidly became haemodynamically unstable due to ongoing bleeding that necessitated emergent surgery to repair the RV apex and intercostal artery.

Recent international guidelines also suggest several recommendations to minimize the risk of cardiac perforation. These include the use of multiple fluoroscopic views, careful deployment of the lead as well as checking for current of injury and extracardiac stimulation.^8,9^ Consideration should also be given to septal lead positioning in those at increased risk of perforation including elderly or female patients with a low body mass index (<20 kg/m^2^).

In conclusion, this case demonstrates an uncommon complication with a cause that was very clear (RV perforation) and another that was only picked up at surgery (intercostal bleed). Patients who present with a haemothorax after pacing should be transferred to a cardiac surgical centre as emergency surgery may be required in some of these patients.

## Supplementary Material

ytac185_Supplementary_Data

## Figures and Tables

**Figure:**
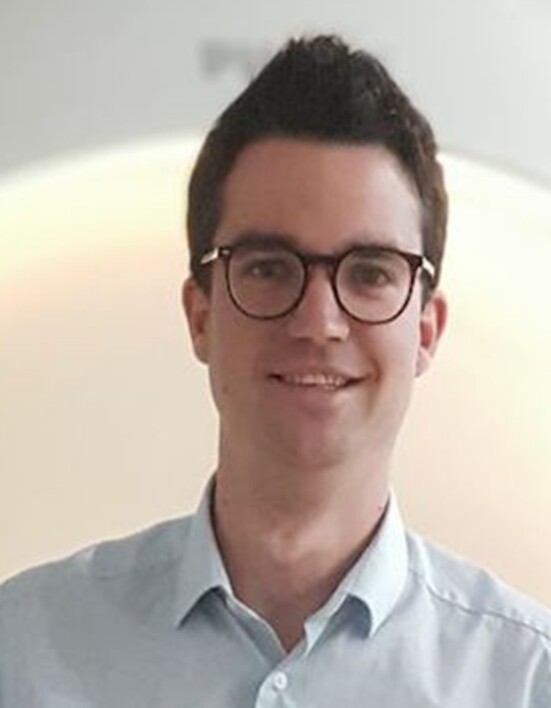


## Lead author biography

Dr Christopher Saunderson, MD, MBChB is a Clinical Fellow in Cardiac Devices at Leeds Teaching Hospitals NHS Trust. He has a keen interest in the fields of cardiac rhythm management and cardiovascular magnetic resonance. Dr Saunderson obtained both Bachelor of Medicine and Doctor of Medicine degrees at Leeds University and completed his Cardiology Training in West Yorkshire.

**Slide sets:** A fully edited slide set detailing these cases and suitable for local presentation is available online as Supplementary data.

**Consent:** The authors confirm that written consent for submission and publication of this case report including images and associated text has been obtained from the patient.

**Funding:** None declared.

## References

[1] Poole JE , Gleva MJ, Mela T, Chung MK, Uslan DZ, Borge R, Gottipaty V, Shinn T, Dan D, Feldman LA, Seide H, Winston SA, Gallagher JJ, Langberg JJ, Mitchell K, Holcomb R. Complication rates associated with pacemaker or implantable cardioverter-defibrillator generator replacements and upgrade procedures: results from the REPLACE registry. Circulation 2010;122: 1553–1561. 20921437 10.1161/CIRCULATIONAHA.110.976076

[2] Matthia E , Matar R, Altshuler E, Kerensky RA, Arnaoutakis G, Shah S, Omar A, Agarwal Z, Miles W, Xiang K. Massive tension hemothorax after pacemaker implantation. Cureus 2021;13:e16754. 34513377 10.7759/cureus.16754 PMC8405410

[3] Nichols J , Berger N, Joseph P, Datta D. Subacute right ventricle perforation by pacemaker lead presenting with left hemothorax and shock. Case Rep Cardiol 2015;2015:983930. 25785204 10.1155/2015/983930 PMC4345244

[4] Carlson MD , Freedman RA, Levine PA. Lead perforation: incidence in registries. Pacing Clin Electrophysiol 2008;31:13–15. 18181903 10.1111/j.1540-8159.2007.00943.x

[5] Akbarzadeh MA , Mollazadeh R, Sefidbakht S, Shahrzad S, Bahrololoumi Bafruee N. Identification and management of right ventricular perforation using pacemaker and cardioverter-defibrillator leads: a case series and mini review. J Arrhythm 2017;33:1–5. 28217220 10.1016/j.joa.2016.05.005 PMC5300868

[6] Sanoussi A , El Nakadi B, Lardinois I, De Bruyne Y, Joris M. Late right ventricular perforation after permanent pacemaker implantation: how far can the lead go? Pacing Clin Electrophysiol 2005;28:723–725. 16008811 10.1111/j.1540-8159.2005.00156.x

[7] Kusumoto FM , Schoenfeld MH, Wilkoff BL, Berul CI, Birgersdotter-Green UM, Carrillo R, Cha Y-M, Clancy J, Deharo J-C, Ellenbogen KA, Exner D, Hussein AA, Kennergren C, Krahn A, Lee R, Love CJ, Madden RA, Mazzetti HA, Moore JC, Parsonnet J, Patton KK, Rozner MA, Selzman KA, Shoda M, Srivathsan K, Strathmore NF, Swerdlow CD, Tompkins C, Wazni O. HRS expert consensus statement on cardiovascular implantable electronic device lead management and extraction. Heart Rhythm 2017;14:e503–e551. 28919379 10.1016/j.hrthm.2017.09.001

[8] Burri H , Starck C, Auricchio A, Biffi M, Burri M, D’Avila A, Deharo J-C, Glikson M, Israel C, Lau C-P, Leclercq C, Love CJ, Nielsen JC, Vernooy K, Dagres N, Boveda S, Butter C, Marijon E, Braunschweig F, Mairesse GH, Gleva M, Defaye P, Zanon F, Lopez-Cabanillas N, Guerra JM, Vassilikos VP, Martins Oliveira M. EHRA expert consensus statement and practical guide on optimal implantation technique for conventional pacemakers and implantable cardioverter-defibrillators: endorsed by the Heart Rhythm Society (HRS), the Asia Pacific Heart Rhythm Society (APHRS), and the Latin-American Heart Rhythm Society (LAHRS). Europace 2021;23:983–1008. 33878762 10.1093/europace/euaa367 PMC12378894

[9] Glikson M , Nielsen JC, Kronborg MB, Michowitz Y, Auricchio A, Barbash IM, Barrabés JA, Boriani G, Braunschweig F, Brignole M, Burri H, Coats AJS, Deharo J-C, Delgado V, Diller G-P, Israel CW, Keren A, Knops RE, Kotecha D, Leclercq C, Merkely B, Starck C, Thylén I, Tolosana JM, ESC Scientific Document Group. ESC Guidelines on cardiac pacing and cardiac resynchronization therapy. Eur Heart J 2021;42:3427–3520.34455430

