# Supplementary material for: An unusual cause of a haemothorax following pacemaker implantation: A case report
Source: Eur Heart J Case Rep. 2022 May 1;6(5):ytac185. doi: 10.1093/ehjcr/ytac185 (PMC9113347; doi:10.1093/ehjcr/ytac185)
Supplement: ytac185_Supplementary_Data [file ytac185_supplementary_data.pptx]

## Slide 1
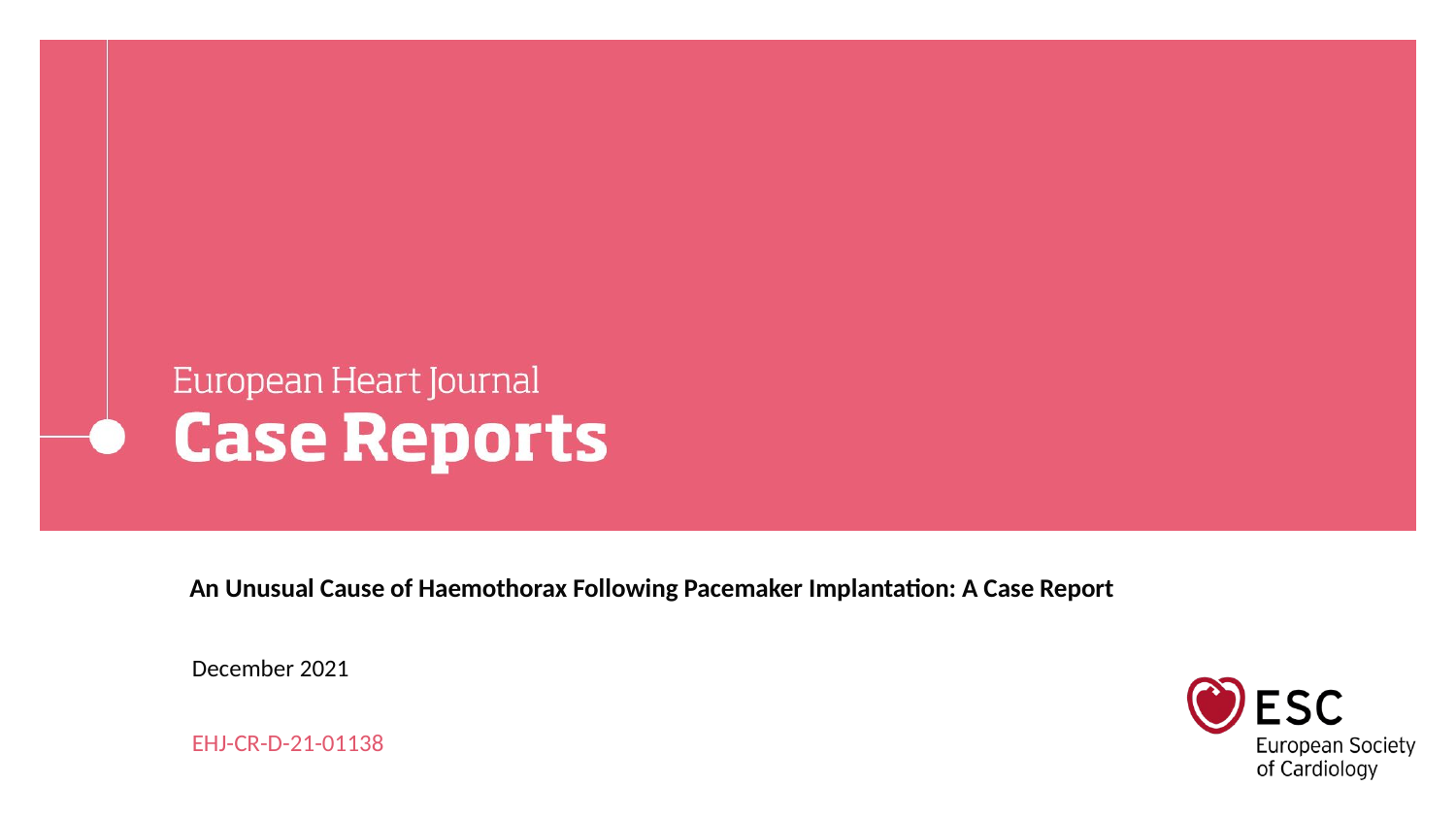

# An Unusual Cause of Haemothorax Following Pacemaker Implantation: A Case Report
December 2021
EHJ-CR-D-21-01138

## Slide 2
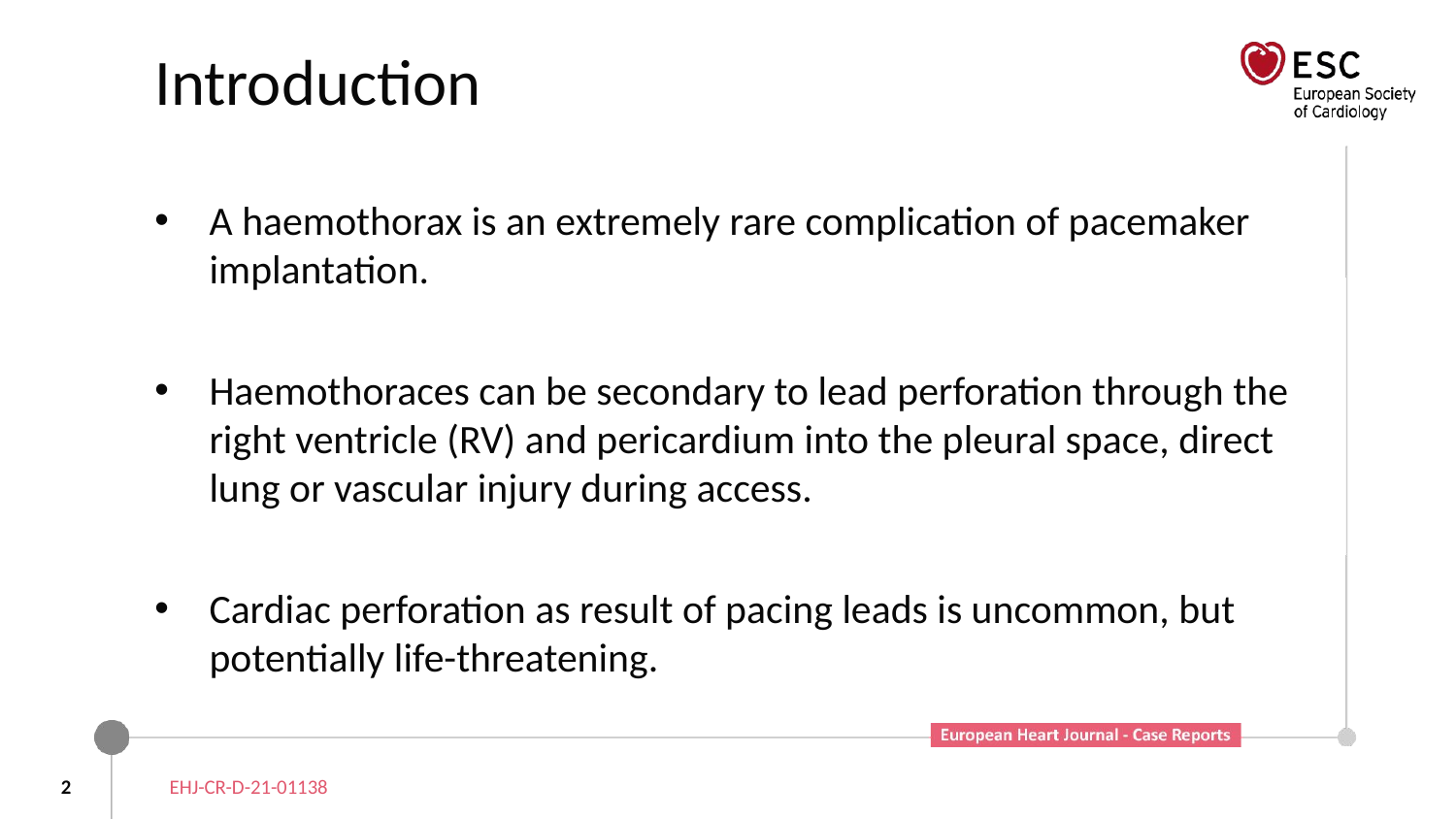

# Introduction
A haemothorax is an extremely rare complication of pacemaker implantation.
Haemothoraces can be secondary to lead perforation through the right ventricle (RV) and pericardium into the pleural space, direct lung or vascular injury during access.
Cardiac perforation as result of pacing leads is uncommon, but potentially life-threatening.
2
EHJ-CR-D-21-01138

## Slide 3
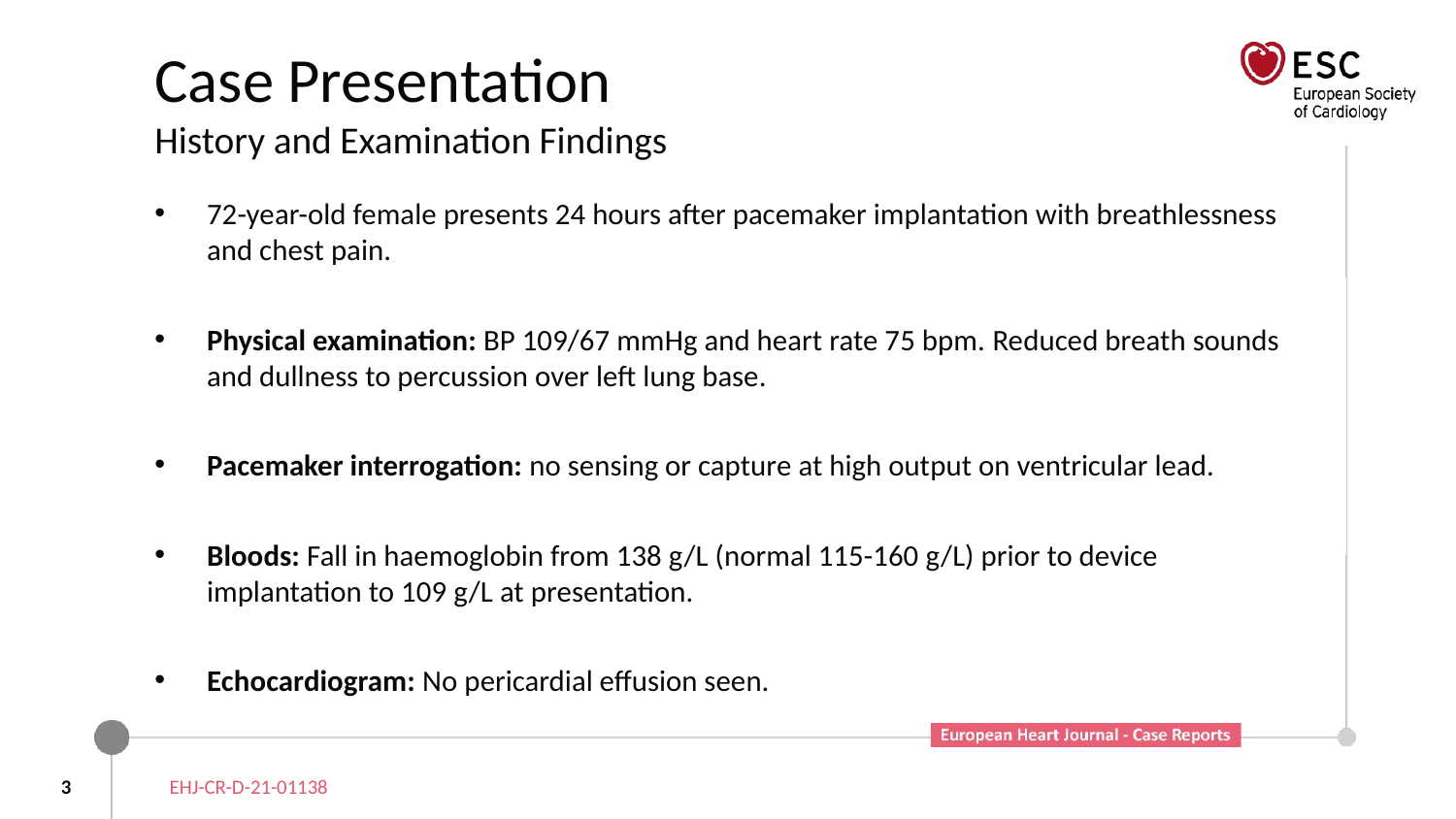

# Case Presentation History and Examination Findings
72-year-old female presents 24 hours after pacemaker implantation with breathlessness and chest pain.
Physical examination: BP 109/67 mmHg and heart rate 75 bpm. Reduced breath sounds and dullness to percussion over left lung base.
Pacemaker interrogation: no sensing or capture at high output on ventricular lead.
Bloods: Fall in haemoglobin from 138 g/L (normal 115-160 g/L) prior to device implantation to 109 g/L at presentation.
Echocardiogram: No pericardial effusion seen.
3
EHJ-CR-D-21-01138

## Slide 4
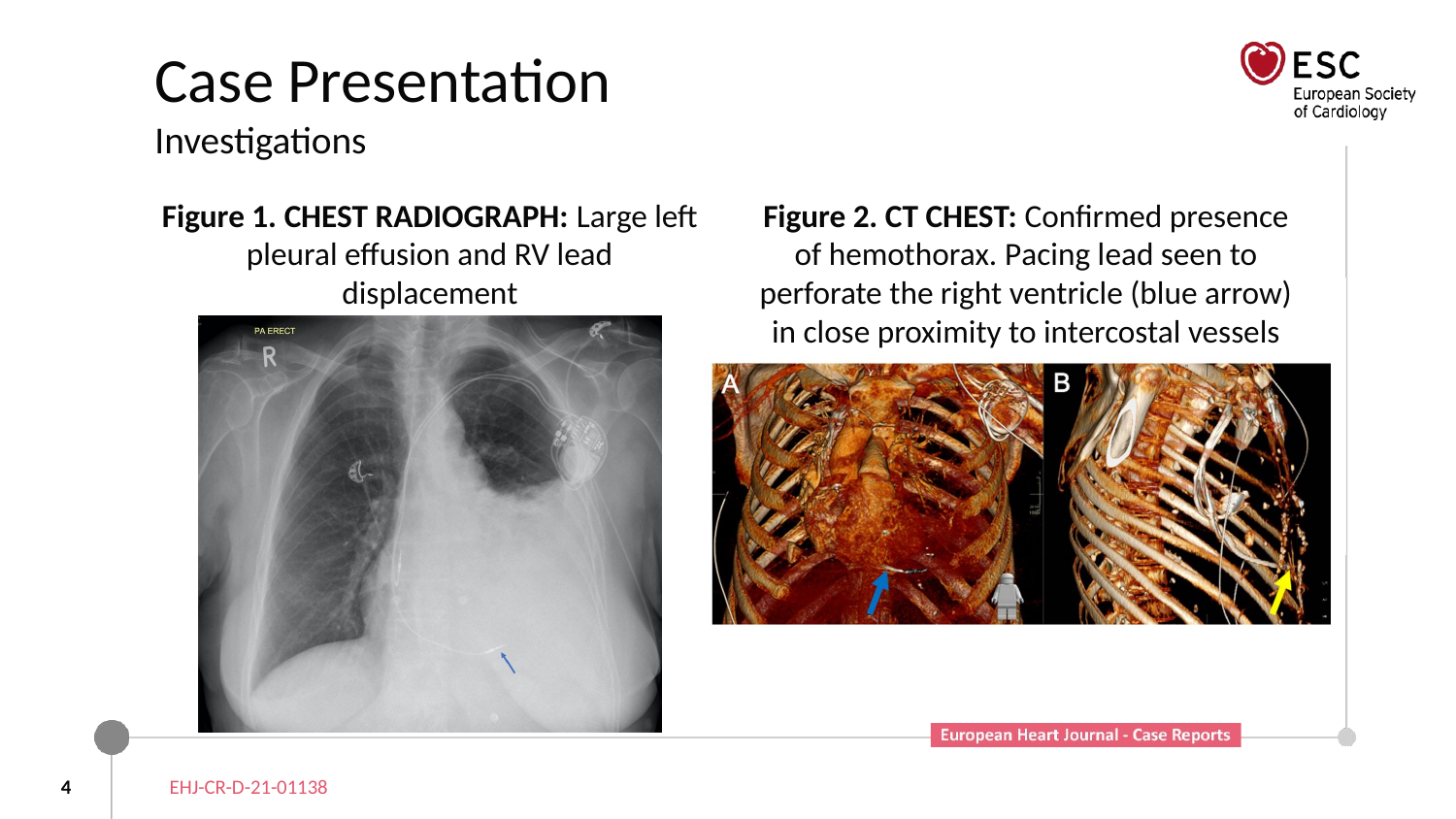

# Case Presentation Investigations
Figure 1. CHEST RADIOGRAPH: Large left pleural effusion and RV lead displacement
Figure 2. CT CHEST: Confirmed presence of hemothorax. Pacing lead seen to perforate the right ventricle (blue arrow) in close proximity to intercostal vessels (yellow arrow)
4
EHJ-CR-D-21-01138

## Slide 5
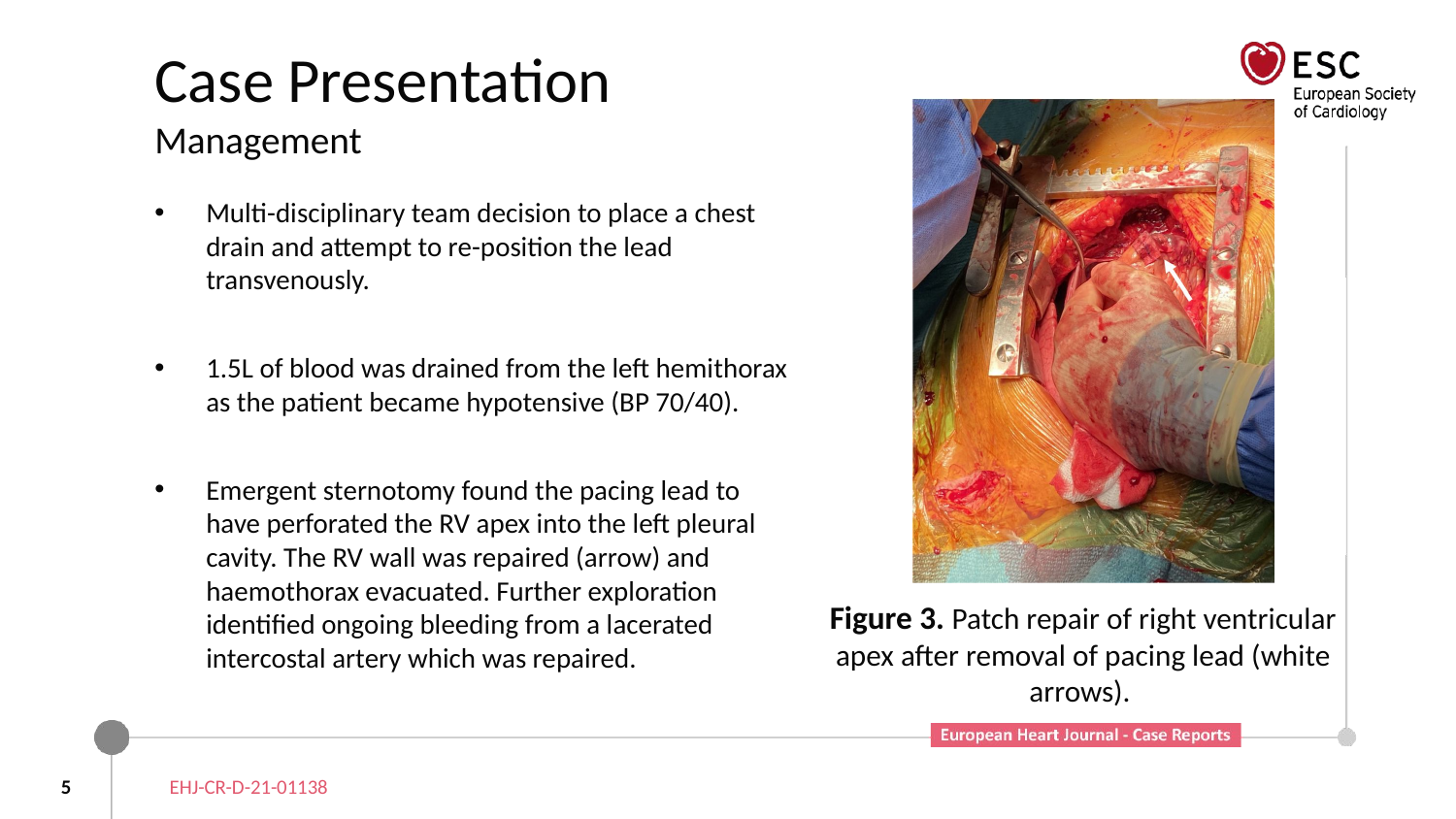

# Case Presentation Management
Multi-disciplinary team decision to place a chest drain and attempt to re-position the lead transvenously.
1.5L of blood was drained from the left hemithorax as the patient became hypotensive (BP 70/40).
Emergent sternotomy found the pacing lead to have perforated the RV apex into the left pleural cavity. The RV wall was repaired (arrow) and haemothorax evacuated. Further exploration identified ongoing bleeding from a lacerated intercostal artery which was repaired.
Figure 3. Patch repair of right ventricular apex after removal of pacing lead (white arrows).
5
EHJ-CR-D-21-01138

## Slide 6
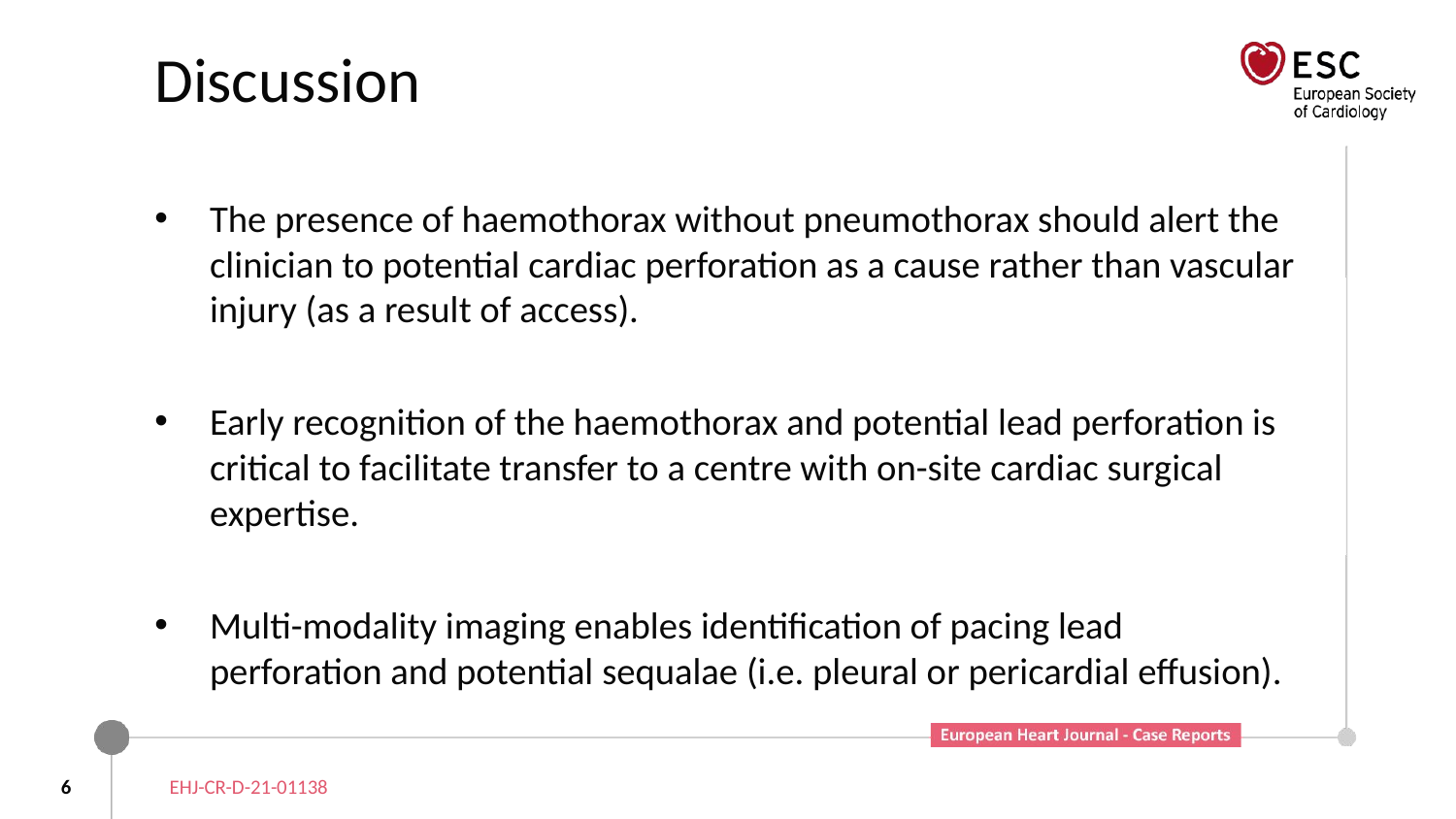

# Discussion
The presence of haemothorax without pneumothorax should alert the clinician to potential cardiac perforation as a cause rather than vascular injury (as a result of access).
Early recognition of the haemothorax and potential lead perforation is critical to facilitate transfer to a centre with on-site cardiac surgical expertise.
Multi-modality imaging enables identification of pacing lead perforation and potential sequalae (i.e. pleural or pericardial effusion).
6
EHJ-CR-D-21-01138

## Slide 7
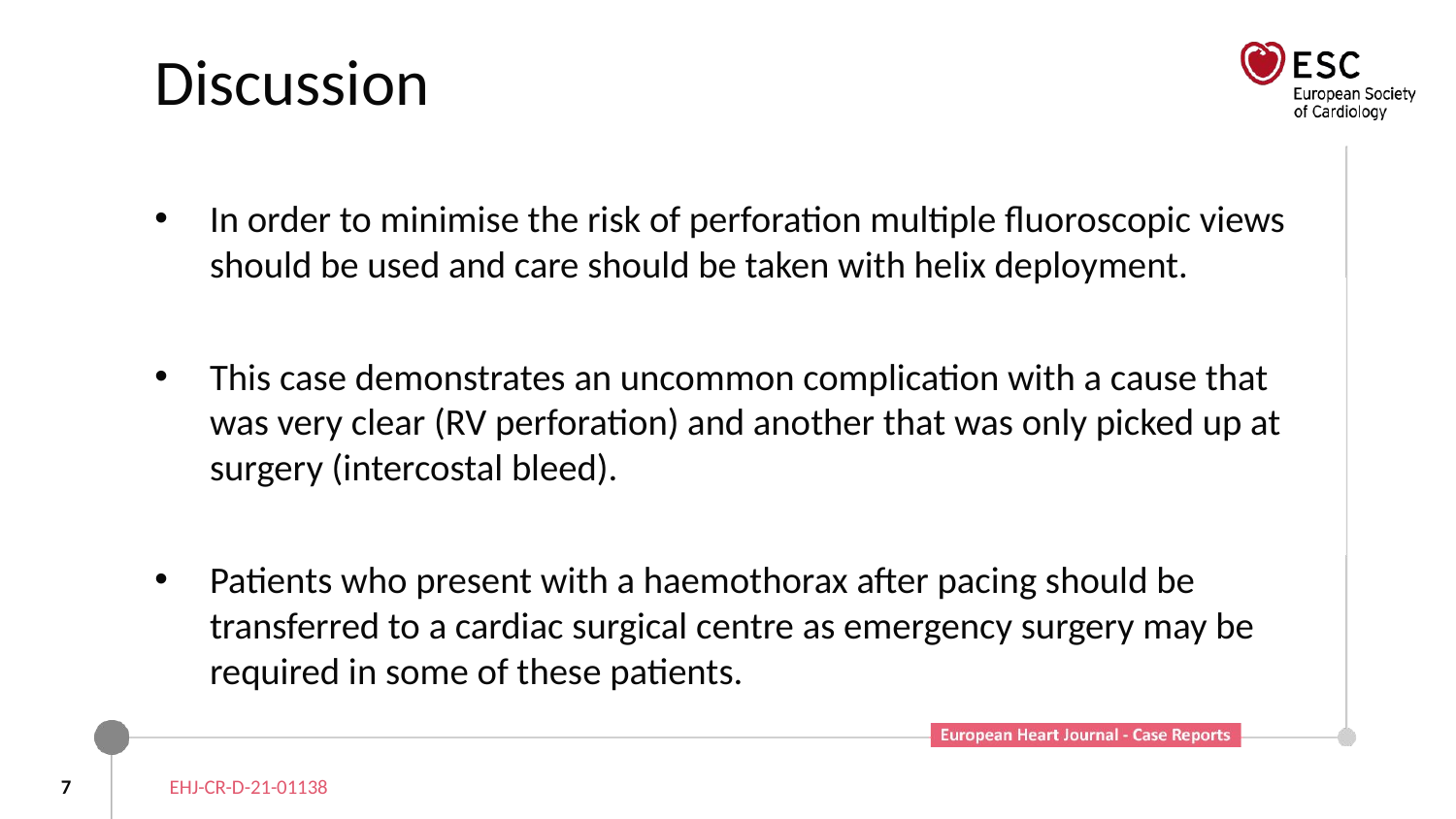

# Discussion
In order to minimise the risk of perforation multiple fluoroscopic views should be used and care should be taken with helix deployment.
This case demonstrates an uncommon complication with a cause that was very clear (RV perforation) and another that was only picked up at surgery (intercostal bleed).
Patients who present with a haemothorax after pacing should be transferred to a cardiac surgical centre as emergency surgery may be required in some of these patients.
7
EHJ-CR-D-21-01138

## Slide 8
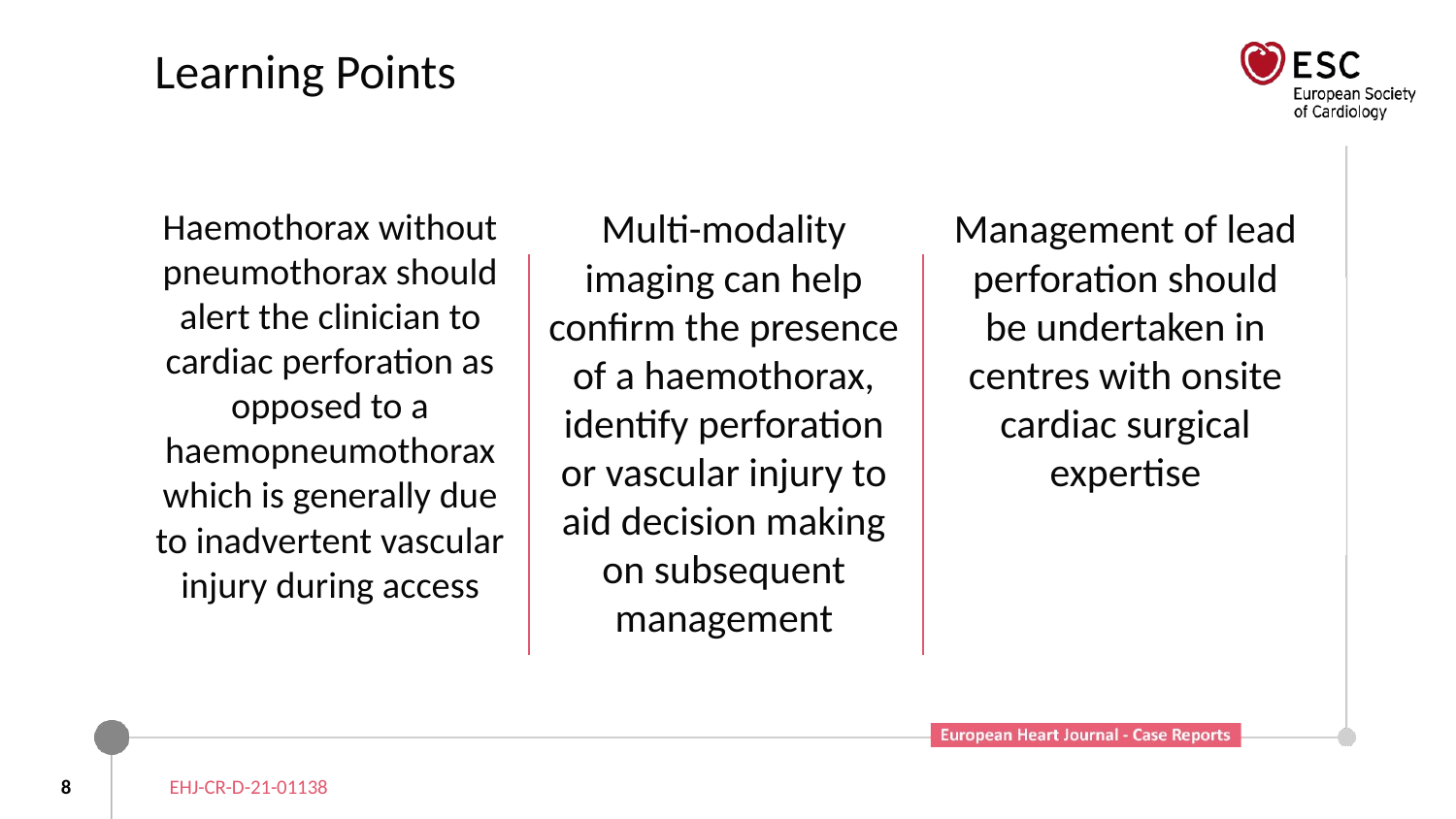

# Learning Points
Haemothorax without pneumothorax should alert the clinician to cardiac perforation as opposed to a haemopneumothorax which is generally due to inadvertent vascular injury during access
Multi-modality imaging can help confirm the presence of a haemothorax, identify perforation or vascular injury to aid decision making on subsequent management
Management of lead perforation should be undertaken in centres with onsite cardiac surgical expertise
8
EHJ-CR-D-21-01138

## Slide 9
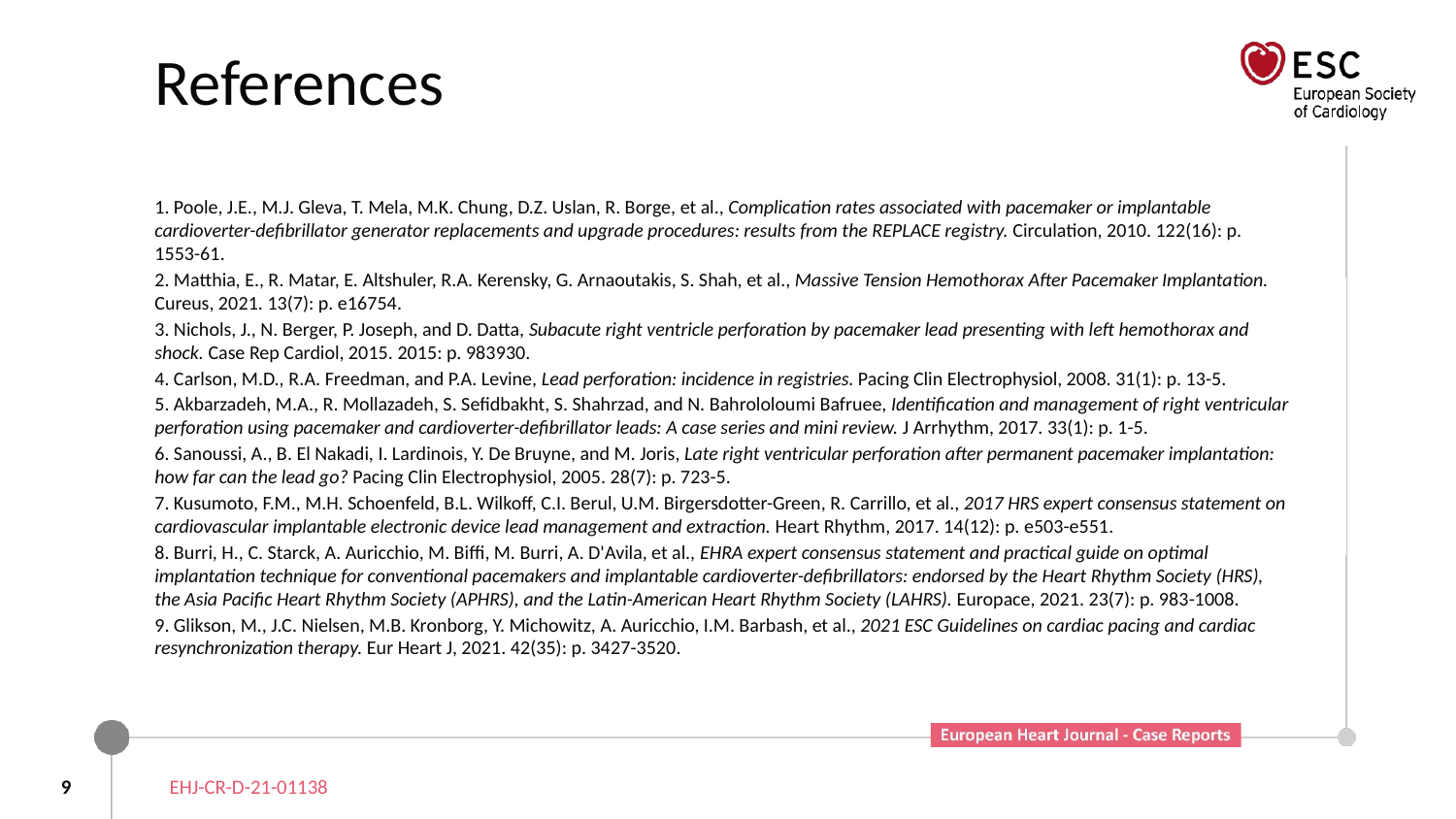

# References
1. Poole, J.E., M.J. Gleva, T. Mela, M.K. Chung, D.Z. Uslan, R. Borge, et al., Complication rates associated with pacemaker or implantable cardioverter-defibrillator generator replacements and upgrade procedures: results from the REPLACE registry. Circulation, 2010. 122(16): p. 1553-61.
2. Matthia, E., R. Matar, E. Altshuler, R.A. Kerensky, G. Arnaoutakis, S. Shah, et al., Massive Tension Hemothorax After Pacemaker Implantation. Cureus, 2021. 13(7): p. e16754.
3. Nichols, J., N. Berger, P. Joseph, and D. Datta, Subacute right ventricle perforation by pacemaker lead presenting with left hemothorax and shock. Case Rep Cardiol, 2015. 2015: p. 983930.
4. Carlson, M.D., R.A. Freedman, and P.A. Levine, Lead perforation: incidence in registries. Pacing Clin Electrophysiol, 2008. 31(1): p. 13-5.
5. Akbarzadeh, M.A., R. Mollazadeh, S. Sefidbakht, S. Shahrzad, and N. Bahrololoumi Bafruee, Identification and management of right ventricular perforation using pacemaker and cardioverter-defibrillator leads: A case series and mini review. J Arrhythm, 2017. 33(1): p. 1-5.
6. Sanoussi, A., B. El Nakadi, I. Lardinois, Y. De Bruyne, and M. Joris, Late right ventricular perforation after permanent pacemaker implantation: how far can the lead go? Pacing Clin Electrophysiol, 2005. 28(7): p. 723-5.
7. Kusumoto, F.M., M.H. Schoenfeld, B.L. Wilkoff, C.I. Berul, U.M. Birgersdotter-Green, R. Carrillo, et al., 2017 HRS expert consensus statement on cardiovascular implantable electronic device lead management and extraction. Heart Rhythm, 2017. 14(12): p. e503-e551.
8. Burri, H., C. Starck, A. Auricchio, M. Biffi, M. Burri, A. D'Avila, et al., EHRA expert consensus statement and practical guide on optimal implantation technique for conventional pacemakers and implantable cardioverter-defibrillators: endorsed by the Heart Rhythm Society (HRS), the Asia Pacific Heart Rhythm Society (APHRS), and the Latin-American Heart Rhythm Society (LAHRS). Europace, 2021. 23(7): p. 983-1008.
9. Glikson, M., J.C. Nielsen, M.B. Kronborg, Y. Michowitz, A. Auricchio, I.M. Barbash, et al., 2021 ESC Guidelines on cardiac pacing and cardiac resynchronization therapy. Eur Heart J, 2021. 42(35): p. 3427-3520.
9
EHJ-CR-D-21-01138
